# Acute Effects of Continuous and Intermittent Blood Flow Restriction on Movement Velocity During Bench Press Exercise Against Different Loads

**DOI:** 10.3389/fphys.2020.569915

**Published:** 2020-11-27

**Authors:** Michal Wilk, Mariola Gepfert, Michal Krzysztofik, Petr Stastny, Adam Zajac, Gregory C. Bogdanis

**Affiliations:** ^1^Institute of Sport Sciences, Jerzy Kukuczka Academy of Physical Education in Katowice, Katowice, Poland; ^2^Department of Sport Games, Faculty of Physical Education and Sport, Charles University, Prague, Czechia; ^3^School of Physical Education and Sport Science, National and Kapodistrian University of Athens, Athens, Greece

**Keywords:** occlusion, resistance exercise, cuff, peak velocity, performance

## Abstract

This study evaluated the effects of continuous and intermittent blood flow restriction (BFR) with 70% of full arterial occlusion pressure on bar velocity during the bench press exercise against a wide range of resistive loads. Eleven strength-trained males (age: 23.5 ± 1.4 years; resistance training experience: 2.8 ± 0.8 years, maximal bench press strength – 1RM = 101.8 ± 13.9 kg; body mass = 79.8 ± 10.4 kg), performed three different testing protocols in random and counterbalanced order: without BFR (NO-BFR); intermittent BFR (I-BFR) and continuous BFR (C-BFR). During each experimental session, subjects performed eight sets of two repetitions each, with increasing loads from 20 to 90% 1RM (10% steps), and 3 min rest between each set. In the C-BFR condition occlusion was kept throughout the trial, while in the I-BFR, occlusion was released during each 3 min rest interval. Peak bar velocity (PV) during the bench press exercise was higher by 12–17% in both I-BFR and C-BFR compared with NO-BFR only at the loads of 20, 30, 40, and 50% 1RM (*p* < 0.001), while performance at higher loads remained unchanged. Mean bar velocity (MV) was unaffected by occlusion (*p* = 0.342). These results indicate that BFR during bench press exercise increases PV and this may be used as an enhanced stimulus during explosive resistance training. At higher workloads, bench press performance was not negatively affected by BFR, indicating that the benefits of exercise under occlusion can be obtained while explosive performance is not impaired.

## Introduction

Athletes as well as recreationally trained individuals are increasingly looking for innovative techniques and methods of resistance training to provide an additional stimulus to break through plateaus, prevent monotony and achieve various training goals (Krzysztofik et al., 2019). Partial or total occlusion of blood flow to the working muscles during resistance exercise, also known as blood flow restriction (BFR), has been used as a complementary training modality, aiming to further increase muscle mass and improve strength (Wilk et al., 2018).

Typically, BFR training involves the use of a tourniquet, an inflatable cuff, or elastic wraps that exert high pressure at the proximal part of the limb (arm or leg), in order to reduce arterial blood flow and to shut venous blood flow during exercise (Takano et al., 2005; Loenneke and Pujol, 2009; Scott et al., 2015). There are different modalities of BFR training, such as continuous BFR during a resistance training set or several sets, intermittent BFR used only during exercise, with release of occlusion during the rest interval, or even occlusion only before exercise, in order to induce ischemic preconditioning (Incognito et al., 2016; Marocolo et al., 2016, 2017, 2018, 2019; Wilk et al., 2020d, e, f). Resistance exercise under BFR affects physiological responses, increases mechanical tension, metabolic stress (Pearson and Hussain, 2015; Teixeira E. L. et al., 2018), cell swelling (Loenneke et al., 2012b), and enhances responses of the endocrine system (Takano et al., 2005; Shimizu et al., 2016). Individual limb characteristics, as well as the width, length, shape and material of the cuffs determine the level of applied pressure and as a consequence, modify post-exercise adaptive responses to BFR training (Loenneke et al., 2012b, 2015; Jessee et al., 2016; Rawska et al., 2019; Wilk et al., 2020e). However, not only physiological, but also mechanical factors related to BFR should be taken into consideration (Wilk et al., 2020d, f). Wilk et al. (2020f) speculated that mechanical work generated by the cuff, in addition to other factors, can potentially augment bar velocity and power output during resistance exercise under BFR. The BFR cuff is a passive element, but during exercise, the strain of the material and the deformation of the cuff may store and return elastic energy, which could affect power output and bar velocity compared to standard conditions (Wilk et al., 2020f).

Despite the fact much attention has been focused on BFR training, only few previous studies have compared the acute effects of BFR during resistance exercise (Luebbers et al., 2014; Teixeira E. L. et al., 2018; Rawska et al., 2019), and only one study has examined its effects on subsequent power output generation (Wilk et al., 2020f). Wilk et al. (2020f) showed that intermittent, high pressure BFR increases bar velocity and power output during the bench press (BP) exercise at 70% one-repetition maximum (1RM). However, only BFR with a wide cuff (10 cm) significantly influenced power output and bar velocity, while no changes were registered with a narrow cuff (4 cm) (Wilk et al., 2020f). As suggested by Wilk et al. (2020f) such potential benefits related to power output and bar velocity can be obtained only under high pressure BFR, but it is still unknown whether these acute gains are influenced by the mode of BFR (e.g., intermittent vs. continuous) or by the magnitude of external load used, relative to maximum strength. Thus, the impact of BFR on the velocity of movement during resistance exercise requires further research.

The BP is one of the most popular upper-body resistance exercises, with numerous variations (e.g., flat, incline, decline, wide-grip, and closed-grip) commonly used in practice (Dunnick et al., 2015; Wilk et al., 2019, 2020c). Given the widespread use of the BP as a basic exercise for developing upper body strength and power output (Stastny et al., 2017), it would be interesting to examine whether the use of continuous or intermittent BFR acutely affects bar velocity during the BP. Furthermore, it would be very useful from a practical viewpoint to determine whether the acute effects of BFR training are modified by the external load used. Therefore, the aim of the present study was to evaluate the effects of continuous and intermittent BFR on bar velocity during the BP exercise at progressive loads, from 20 to 90% 1RM. It was hypothesized that both continuous and intermittent BFR would increase bar velocity during the BP at all used loads.

## Materials and Methods

**FIGURE 1:**
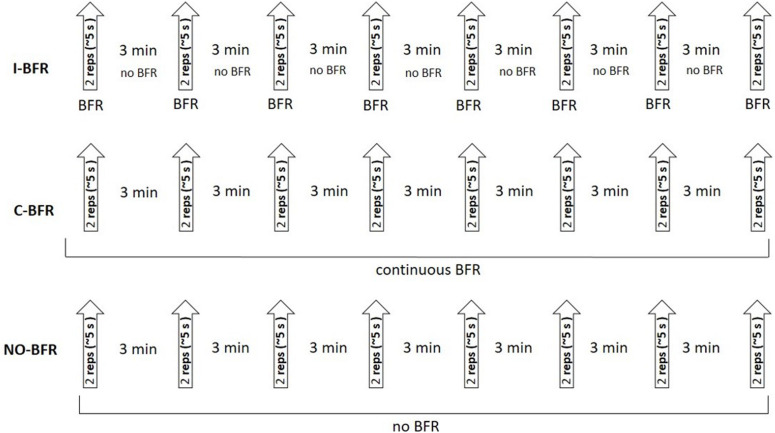
Schematic representation of the experimental protocol. I-BFR, intermittent blood flow restriction; C-BFR, continuous blood flow restriction; NO-BFR, no blood flow restriction (control condition).

The experiment was performed following a randomized crossover design, where each subject performed three different testing protocols in random and counterbalanced order, 1 week apart: without BFR (NO-BFR); intermittent BFR (I-BFR) and continuous BFR (C-BFR). Before the main tests, three familiarization session were performed. One week before the first main session, maximal BP strength (one repetition maximum-1RM) was evaluated. During each experimental session, subjects performed eight sets of two repetitions each, with increasing loads from 20 to 90% 1RM (10% steps), and 3 min rest between each set. Each repetition was performed with a 2 s duration of the eccentric phase of the movement and a maximal tempo in the concentric phase of the BP exercise. In the C-BFR condition occlusion was kept throughout the trial, while in the I-BFR, occlusion was released during each 3 min rest interval (Figure 1). The following variables were measured using a linear position transducer: PV and MV. All testing sessions were performed in the Strength and Power Laboratory at the Academy of Physical Education in Katowice, Poland.

### Subjects

Power analysis indicated a minimum sample size of nine participants would be needed to detect an effect size (ES) of 0.44. This value was obtained from a recent study examining the acute effects of BFR on movement velocity during the bench press (Wilk et al., 2020d). Power analysis was performed using the following parameters: type of analysis was set to repeated measures ANOVA, the required power was set to 0.80, alpha was set to 0.05, and the correlation between repeated measures was set to *r* = 0.5 (G^∗^Power software, v.3.1.9.2).

Eleven healthy men with experience in resistance training (2.8 ± 0.8 years) volunteered for the study after completing an informed consent form (age = 23.5 ± 1.4 years; body mass = 79.8 ± 10.4 kg; BP 1RM = 101.8 ± 13.9 kg). The main inclusion criteria were: a BP personal best of at least 120% body mass and that the subject was free from musculoskeletal injuries for at least 6 months before the study. The subjects were instructed to maintain their normal dietary habits over the course of the study for the duration of the experiment. The participants were instructed to maintain their usual hydration and dietary habits and not to use any supplements or stimulants during the study period and registering their calorie intake using “MyFitnessPal” software (Teixeira V. et al., 2018) every 24 h before the testing procedure. Compliance of these variables was verified at the start of each visit, before any data collection. They were informed about the benefits and potential risks of the study before providing their written informed consent for participation, and were allowed to withdraw from the study at any time. The study protocol was approved by the Bioethics Committee for Scientific Research, at the Academy of Physical Education in Katowice, Poland (02/2019), and all procedures were in accordance with the ethical standards of the Declaration of Helsinki, 1983.

### Procedures

#### Familiarization Session and the 1RM Strength Test

Two weeks before the main experiment, the subjects performed at three familiarization sessions. During the familiarization sessions, each subject performed five sets of two repetitions of the BP under BFR against a load of 30, 50, and 70% of their estimated 1RM. The familiarization sessions were performed in order to minimize possible learning effects during the main tests. One week before the main experiment the 1RM BP test was performed. On arrival, body mass was measured and then the subjects cycled on a cycle-ergometer for 5 min, followed by a general upper body warm-up as described elsewhere (Wilk et al., 2019). Then, the subjects performed 15, 10, and 5 BP repetitions using 20, 40, and 60% of their estimated 1RM. The first testing load was set to an estimated 80% 1RM, and was increased by 2.5–10 kg for each subsequent attempt. This process was repeated until failure. During the 1RM test, the subjects executed one repetition with 2 s of duration in the eccentric phase and maximal speed in the concentric phase of movement. According to guidelines by Wilk et al. (2020a, b, c) the 1RM test was performed with the same tempo of movement as all experimental trials. The rest interval between successful trials was 5 min. Hand placement on the barbell was set at 150% of the individual bi-acromial distance, and this was used for all main trials.

#### Experimental Sessions

In a randomized and counterbalanced order, the subjects performed the BP exercise under three different testing conditions: without BFR (NO-BFR); intermittent BFR (I-BFR) and continuous BFR (C-BFR). During each testing protocol the subject performed the BP against an individualized load, starting from 20 to 90% 1RM, which was increased progressively by 10% in each subsequent set (for a total of eight sets) with a 3 min rest interval between sets. During each set the subjects performed two repetitions, with a 2 s duration of the eccentric phase and maximal velocity in the concentric phase of movement. Every repetition was performed without bouncing the barbell off the chest, and without intentionally pausing at the transition between the eccentric and concentric phases. A linear position transducer system (Tendo Power Analyzer, Tendo Sport Machines, Trencin, Slovakia) was used for the evaluation of bar velocity (Garnacho-Castaño et al., 2015). Measurements were made independently for each repetition and automatically converted into values of bar velocity. PV was obtained from the best repetition performed in a particular set. The MV was obtained as the mean of two repetitions performed in particular sets. All subjects completed the described testing protocol that was carefully replicated in subsequent experimental sessions.

### Blood Flow Restriction

During the BFR sessions, subjects wore pressure cuffs at the most proximal region of both arms. For this experiment we used KAATSU cuffs (Master, Sato Sports Plaza, Tokyo, Japan), which are characterized as “narrow” 4-cm cuffs (Wilk et al., 2020f). To determine the individual pressure value, after a 5 min rest interval, the value of full arterial occlusion pressure was determined. The measurement was conducted twice on each limb and the obtained differences were within 20 mmHg, with the average value used to set the cuff pressure for the exercise protocol (Wilk et al., 2020f). The cuff pressure for the BP exercise was set to ∼70% of full arterial occlusion pressure (231 ± 20 mmHg). The level of vascular restriction was monitored using a handheld Edan SD3 Doppler with an OLED screen and a 2 mHz probe made by Edan Instruments (Shenzhen, China). For the I-BFR protocol, the occlusion was applied immediately before the start of the exercise and released upon completion of the second repetition. Thus, in the I-BFR condition, the occlusion was released during each 3 min rest interval. The occlusion for I-BFR lasted approximately 40 s (eight sets, ∼5 s of effort for each set). For the C-BFR condition the occlusion was applied 1 min before the start of the first set of the BP, and was maintained for all experimental sets, and also during the rest intervals. The occlusion for C-BFR lasted approximately 23 min (eight sets, 3 min rest intervals, ∼5 s of effort for each set).

### Statistical Analysis

All statistical analysis were performed using Statistica 9.1. Results are presented as means with standard deviations. The Shapiro-Wilk, Levene, and Mauchly’s tests were used in order to verify the normality, homogeneity and sphericity of the sample data variances, respectively. Differences between the NO-BFR, I-BFR and C-BFR conditions were examined using repeated measures two-way ANOVA (3 conditions × 8 loads). The statistical significance was set at *p* < 0.05. ESs for main effects and interactions were determined by partial eta squared (η^2^). Partial eta squared values were classified as small (0.01–0.059), moderate (0.06–0.137) and large (>0.137). *Post hoc* comparisons using the Tukey’s test were conducted to locate the differences between mean values when a main effect or an interaction was found. For pairwise comparisons, ESs were determined by Cohen’s d which was characterized as large (*d* > 0.8), moderate (*d* between 0.8 and 0.5), small (*d* between 0.49 and 0.20) and trivial (*d* < 0.2) (Cohen, 1988). Percent changes with 90% confidence intervals (90CI) were also calculated. Statistical significance was set at *p* < 0.05.

## Results

**FIGURE 2:**
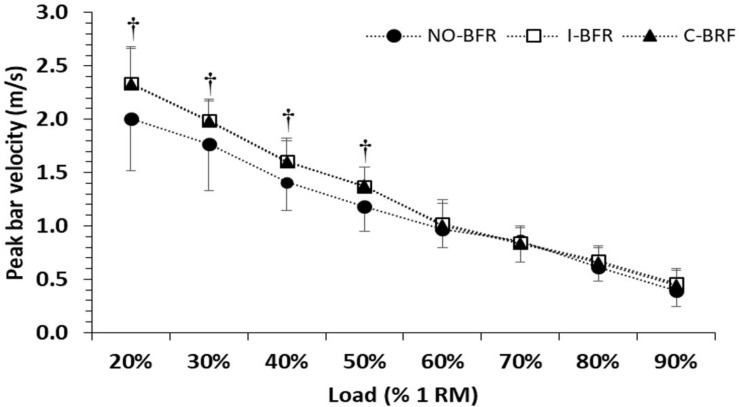
Peak bar velocity during the bench press exercise for eight different loads. 1RM, 1 repetition maximum; NO-BFR, no blood flow restriction (control); I-BFR, intermittent blood flow restriction; C-BFR, continuous blood flow restriction.

The two-way repeated measures ANOVA showed statistically significant interaction for PV (conditions × load; *p* < 0.0001; η^2^ = 0.33; Table 1). The *post hoc* analysis revealed that PV was higher by 12–17% in both I-BFR and C-BFR compared with NO-BFR at the loads of 20, 30, 40, and 50% 1RM (Table 1 and Figure 2). For example, at the lowest load (20% 1RM), PV in the NO-BFR condition was 2.01 ± 0.49 m/s (90CI; 1.77–2.25 m/s), and was increased to 2.34 ± 0.34 m/s (90CI; 2.17–2.51 m/s; *p* < 0.0001; *d* = 0.82) in the I-BFR and to 2.33 ± 0.33 m/s (90CI; 2.17–2.49 m/s; *p* < 0.0001; *d* = 0.80) in the C-BFR condition. At the load of 50% 1RM, PV in the NO-BFR condition was 1.18 ± 0.23 m/s (90CI; 1.07–1.29 m/s), and was increased to 1.37 ± 0.18 m/s (90CI; 1.28–1.46 m/s; *p* < 0.0001; *d* = 1.04) in the I-BFR and to 1.38 ± 0.17 m/s (90CI; 1.30–1.46 m/s; *p* < 0.0001; *d* = 0.80) in the C-BFR condition. No differences were found between the I-BFR and C-BFR conditions at any load.

**TABLE 1:** Peak bar velocity during the bench press exercise for eight different loads.

	Load (% 1RM)								
	20% 1RM	30% 1RM	40% 1RM	50% 1RM	60% 1RM	70% 1RM	80% 1RM	90% 1RM	
		
Peak bar velocity (m/s)									*p* (condition × load)
NO-BFR	2.01 ± 0.49 (1.77–2.25)	1.77 ± 0.44 (1.55–1.99)	1.41 ± 0.27 (1.28–1.54)	1.18 ± 0.23 (1.07–1.29)	0.97 ± 0.17 (0.89–1.05)	0.86 ± 0.20 (0.76–0.96)	0.61 ± 0.13 (0.55–0.67)	0.39 ± 0.15 (0.32–0.46)	0.0001
I-BFR	2.34 ± 0.34 (2.11–2.57)	1.99 ± 0.20 (1.86–2.13)	1.61 ± 0.21 (1.47–1.75)	1.37 ± 0.18 (1.25–1.49)	1.02 ± 0.23 (0.86–1.17)	0.84 ± 0.16 (0.73–0.94)	0.67 ± 0.14 (0.57–0.76)	0.46 ± 0.14 (0.37–0.56)	
C-BFR	2.33 ± 0.33 (2.17–2.49)	1.98 ± 0.19 (1.89–2.07)	1.60 ± 0.20 (1.50–1.70)	1.38 ± 0.17 (1.30–1.46)	1.00 ± 0.21 (0.90–1.10)	0.83 ± 0.15 (0.76–0.90)	0.66 ± 0.14 (0.59–0.73)	0.44 ± 0.14 (0.37–0.51)	

*Results are mean ± SD (90% confidence intervals). 1RM, 1 repetition maximum; NO-BFR, no blood flow restriction (control); I-BFR, intermittent blood flow restriction; C-BFR, continuous blood flow restriction.*

There was also a main effect for condition for PV (*p* = 0.001; η^2^ = 0.50). The *post hoc* analysis showed that PV during both C-BFR and I-BFR conditions was higher compared to NO-BFR (*p* = 0.002 and *p* = 0.004, respectively).

There was no significant condition × load interaction (*p* = 0.342) or main effect (*p* = 0.871) for conditions for MV (Table 2).

**TABLE 2:** Mean bar velocity during the bench press exercise for eight different loads.

	Load (% 1RM)								
	20% 1RM	30% 1RM	40% 1RM	50% 1RM	60% 1RM	70% 1RM	80% 1RM	90% 1RM	
		
	Mean bar velocity (m/s)								*p* (condition × load)
NO-BFR	1.41 ± 0.24 (1.29–1.53)	1.22 ± 0.14 (1.15–1.29)	1.05 ± 0.12 (0.99–1.11)	0.90 ± 0.08 (0.86–0.94)	0.70 ± 0.11 (0.65–0.75)	0.55 ± 0.10 (0.50–0.60)	0.39 ± 0.08 (0.35–0.43)	0.24 ± 0.04 (0.22–0.26)	0.342
I-BFR	1.40 ± 0.17 (1.32–1.48)	1.25 ± 0.12 (1.19–1.31)	1.02 ± 0.09 (0.98–1.06)	0.86 ± 0.07 (0.83–0.89)	0.72 ± 0.06 (0.69–0.75)	0.64 ± 0.08 (0.60–0.68)	0.40 ± 0.07 (0.37–0.43)	0.23 ± 0.06 (0.20–0.26)	
C-BFR	1.47 ± 0.17 (1.39–1.5)	1.28 ± 0.13 (1.22–1.34)	1.06 ± 0.10 (1.01–1.11)	0.87 ± 0.15 (0.80–0.94)	0.69 ± 0.14 (0.62–0.76)	0.57 ± 0.09 (0.53–0.61)	0.42 ± 0.09 (0.38–0.46)	0.27 ± 0.08 (0.23–0.31)	

*Results are mean ± SD (90% confidence intervals). 1RM, 1 repetition maximum; NO-BFR, no blood flow restriction (control); I-BFR, intermittent blood flow restriction; C-BFR, continuous blood flow restriction.*

## Discussion

The main finding of the study was that both intermittent and continuous BFR significantly increased PV during the BP exercise for lighter loads (20–50% 1RM) but not for higher loads 60–90% 1RM, partially confirming our hypothesis. Furthermore, the results of the present study did not reveal any significant influence of BFR on MV at any load. Therefore, the results of this study indicate that BFR using a narrow cuff, commonly used for this type of training (KAATSU cuff) enhance peak velocity of the bar when the load is light, while they have no negative effect on PV or MV at any load, thus allowing the use of a wide range of resistances, without performance reduction in the first two repetitions.

Currently, only one previous study has analyzed the acute impact of BFR on bar velocity during the BP exercise, using a single load of 70% 1RM and two types of cuffs: a wider 10 cm cuff and a narrow 4 cm cuff (Wilk et al., 2020f), identical to that used in the present study. The findings of study Wilk et al. (2020f) showed that bar velocity was increased but only when the wide cuff was used, while no changes in any BP performance were seen when the narrow cuff was used at 70% 1RM. In that respect, the results of the present study, are consistent with the findings of that study regarding the load 70% 1RM and the cuff width used. As previously suggested, the acute improvement of performance may be obtained only when wider BFR cuffs with high pressure are used during exercise (Wilk et al., 2020d), but it is unknown whether this effect is purely mechanical or if it also involves other physiological acute responses (Loenneke J. et al., 2012; Rossow et al., 2012). In the present study PV, but not MV, was enhanced in the lower resistive loads (up to 50% 1RM), despite the fact that the cuff was narrow and cuff pressure was set to 70% AOP.

The lack of performance enhancement when resistance was ≥60% 1RM, may be explained by the possibility of an equally compromised blood flow during exercise and rest interval in all conditions, irrespective of external pressure. When lifting loads at or above 60% 1RM, intramuscular pressure is high due to muscle tension, and thus blood flow during exercise is occluded (Sjøgaard et al., 1988; Golas et al., 2015). Also, blood flow during recovery in the latter four sets in all trials, may be compromised due to muscle swelling, as a result of osmotic changes and fluid shifts into the muscle (Sjøgaard et al., 1988; Ploutz-Snyder et al., 1995; Bell et al., 2020). Thus, if blood flow during exercise and recovery is reduced due to muscle tension and muscle swelling, as may be the case in heavier loads, then an additional BFR by the cuffs would not cause any further metabolic effects, explaining the similar performance in all three conditions when the load was ≥60% 1RM. In contrast, when the loads are very low, there may be less blood flow decrease due to muscle contraction and less muscle swelling during recovery, and thus the effects of external pressure may differentiate the metabolic responses in both I-BFR and C-BFR, compared with the control condition. In a previous study (Wilk et al., 2020f), it was shown that at higher resistive loads (70% 1RM) a higher occlusion pressure and a wider cuff are necessary to achieve an increase in bar velocity during exercise under BFR. Therefore, it can be speculated that when the external load increases in order to induce greater bar velocity under BFR, a simultaneous increase in pressure or width of the cuff must occur or both at the same time. Therefore, it may be suggested that there may be an interaction between resistive load, cuff width and cuff pressure, which determines the magnitude of acute changes in performance during exercise under BFR, which is also consistent with previous studies (Loenneke et al., 2012b; Rossow et al., 2012).

A possible explanation for the increases in PV of the bar for both I-BFR and C-BFR, may be ischemic preconditioning (de Souza et al., 2019). Ischemic preconditioning is a non-invasive technique inducing transient peripheral hypoxia and subsequently enhancing tissue tolerance against ischemia–reperfusion (Paradis-Deschênes et al., 2016). The ischemic preconditioning technique has been suggested as a potential ergogenic aid to improve exercise performance (Incognito et al., 2016; Marocolo et al., 2016, 2017). The hyperemia experienced following occlusion is related to increased nitric oxide production (Singh et al., 2017). Furthermore, an increased phosphocreatine resynthesis, altered oxy-deoxyhemoglobin kinetics (Bailey et al., 2012), and increased oxygen consumption (Andreas et al., 2011) following brief ischemia, may all play a significant role in improving subsequent exercise performance. However, the effects of previous ischemia would be expected to be enhanced in each subsequent set, which was not the case in the present study, as improvements in PV were observed in only the first four loads. An additional factor which may influence peak performance in both I-BFR and C-BFR may be related with the bidirectional brain-body integration mechanism, which may promote physiological responses through mechanical-sensory receptors (Taylor et al., 2010; Cromwell and Panksepp, 2011; de Souza et al., 2019) thus increasing resistance exercise performance (de Souza et al., 2019). Such physiological responses may potentially explain the significant increase in PV for conditions under BFR compared to NO-BFR.

The most likely factor that may have enhanced PV in the present study in both BFR conditions is the mechanical energy generated by the cuff (Rawska et al., 2019; Wilk et al., 2020d, f). A cuff is a passive element, but during the eccentric phase of the movement, the strain of the material of the cuff, can store and return elastic energy during the concentric phase of the movement (Harman and Frykman, 1990; Wilk et al., 2020f). This effect may be similar with the phenomenon of enhanced mechanical energy when compressive gear (special shirt for example Inzer or Titan) is used during bench press or powerlifting competitions (Harman and Frykman, 1990), where the energy stored in these elements during the eccentric phase is returned during the lifting phase, resulting in a “rebound” effect. This may be a possible explanation for the increases in PV, but not MV, during fast movements against lower resistive loads. When the load is higher, movement is much slower (see Figure 2), and therefore a wider and highly pressurized cuff which may store much more energy, is needed to induce performance changes (Wilk et al., 2020f). Confirmation of the importance of cuff width and cuff pressure in order to induce performance changes has been provided in the study of Wilk et al. (2020d), who showed a significant acute increase in maximum strength in BP when a wide cuff with pressure of 150% AOP, but not with 100% AOP, was used for BFR.

In the present study there were no significant differences between I-BFR and C-BFR conditions regarding performance enhancement (Figure 2 and Tables 1, 2). The lack of differences between the I-BFR and C-BFR is surprising, since there was a large difference in duration of time under occlusion between both conditions. The occlusion during the BP with C-BFR lasted approximately 23 min (eight sets, 3 min rest intervals, ∼5 s of effort for each set), however, during I-BFR it was only approximately 40 s (eight sets, ∼5 s of effort for each set). Previous studies showed that C-BFR leads to the immediate onset of physiological and metabolic stress (Neto et al., 2018; Wilk et al., 2018; Okita et al., 2019), which consequently may lead to increased fatigue and decreased exercise performance compared to NO-BFR condition (Wernbom et al., 2009; Loenneke et al., 2012a). However, it is expected that due to the brief duration of occlusion the I-BFR, metabolic disturbances would be much lower compared to C-BFR (Pearson and Hussain, 2015; Teixeira E. L. et al., 2018; Okita et al., 2019). Thus, despite the possible differences in metabolic stress between I-BFR and C-BFR, changes in PV were identical, suggesting that enhancement of PV should be attributed to mechanical rather to physiological factors.

Despite the greater metabolic stress potentially induced by C-BFR (Okita et al., 2019), the results did not show any negative effects on PV and MV at any load, compared with NO-BFR (Figure 2 and Tables 1, 2). This may be caused by the short duration of each set (two repetitions lasting approximately 5 s) and the fact that such brief efforts are fueled mainly by phosphocreatine (Bird et al., 2005), which is largely restored during short rest intervals (Bogdanis et al., 1996; Dawson et al., 2007). Performing repetitions to exhaustion at each load would be expected to cause significant reductions in exercise capacity under BFR (Wernbom et al., 2009). In contrast, peak performance is either enhanced at lower loads, or remains unaffected by BFR at higher loads. However, attention should be paid not only to the direct effect of BFR on exercise capacity but also on the potential post-exercise physiological consequences, such as possible muscle damage or even rhabdomyolysis (Wernbom et al., 2019, 2020). Exercise-induced muscle damage is typically caused by unaccustomed eccentric exercise with high load, while BFR in combination with resistance exercise may cause muscle damage, even when exercising against low loads to volitional exhaustion for multiple sets (Yasuda et al., 2015; Sieljacks et al., 2016). Possible muscle damage after exercise under BFR will affect the process of recovery and may affect the quality of the training sessions that follow. Unfortunately, in the present study we did not assess muscle damage indices in the days following the exercise sessions, since this was not within the scope of this research. However, load volume was low, (eight sets of two repetitions each) and exercise was not exhaustive, as the recovery interval was full (3 min). Moreover, our subjects did not report any signs of muscle damage, such as delayed onset muscle soreness. Nevertheless, the C-BFR condition, where blood flow is restricted for 23 min, may result in muscle damage even with this relatively low volume workout (Wernbom et al., 2019, 2020). Thus, the I-BFR seems more attractive in order achieve improved bench press performance and thus training load, while minimizing the negative effects of BFR.

Although the results of the present study showed that both intermittent and continuous BFR during resistance exercise may be used to enhance peak performance during upper body resistance exercises, there are certain limitations which should be addressed. Although the results showed that both I-BFR as well C-BFR impact bar velocity, during the BP at light and moderate loads, the causes of these changes could not be determined and explained due to the lack of physiological and biomechanical evaluations, which could provide possible explanations. Furthermore, the results of this study may not translate to other types of exercises, different loads or different cuff pressures, and thus further research is required.

### Practical Implications

The present study showed that resistance exercise under narrow BFR can be effectively used to increase bar velocity during resistance exercises of the upper body. However, the observed increases in bar velocity apply only to peak values and light to moderate external loads. The applied BFR techniques using a narrow cuff, do not seem to improve MV of the bar and are not effective with loads over 50% 1RM. As previously shown, greater external loads require a higher pressure or wider cuffs to induce a more severe metabolic stress and increase bar velocity. Furthermore, despite the lack of an increase in PV following BFR at higher loads it should be indicated that C-BFR during resistance training could induce additional physiological responses (not evaluated in the present study), such as metabolic stress, endocrine responses, reinforcement of intramuscular signaling, and increased recruitment of fast twitch muscle fibers. Therefore, maintaining mean and peak bar velocity while increasing physiological responses during resistance exercise under BFR, can be a significant factor determining the level of post-exercise adaptive changes. Despite the beneficial effect of BFR on acute bar velocity changes, occlusion can also cause several side effects, especially when used too frequently. The possibility of muscle damage following this protocol cannot be excluded, especially in the C-BFR condition, where blood flow is restricted for a long time. Thus, the I-BFR may be preferable in order to maximize training effects, without the negative effects of BFR.

Further the disadvantages relate primarily to the weakening of the musculature in the area of direct application of the occlusive cuff, especially when the cuff is narrow (Kacin and Strazar, 2011; Ellefsen et al., 2015). Therefore, therapists and trainers should introduce BFR protocols carefully and gradually progress them over time, to ensure that protective adaptations (i.e., a repeated bout effect) can take place in order to minimize the risk of excessive muscle stress and damage (Clark and Manini, 2017; Wernbom et al., 2019). Also, the intermittent form of BFR may be preferable. Therefore, the use of BFR during resistance exercise should be used as a supplementary and not a main method to enhance adaptive responses. It seems that a combination of heavy loaded traditional resistance exercise and supplementary exercise under BFR provide the most desired adaptive changes.

## Conclusion

The results of the present study indicated that BFR used during resistance exercise increases peak bar velocity and thus can by useful for improving explosive performance during resistance training. However, such improvements under BFR were observed only at loads from 20 to 50% 1RM and did not carry over to higher loads. Peak velocity enhancement under BFR may be explained by both metabolic and mechanical factors, with the latter possibly being more influential. These findings expand the scientific knowledge related to the acute effects of BFR during resistance exercise, which also has significant practical implications.

## Data Availability Statement

The raw data supporting the conclusions of this article will be made available by the authors, without undue reservation.

## Ethics Statement

The studies involving human participants were reviewed and approved by The study protocol was approved by the Bioethics Committee for Scientific Research, at the Academy of Physical Education in Katowice, Poland (02/2019). The patients/participants provided their written informed consent to participate in this study.

## Author Contributions

MW, MG, PS, and MK: study conception and design. MW and MG: acquisition of data. MW, MK, and PS: analysis and interpretation of data. MW, MG, GB, and PS: drafting of manuscript. MW, PS, AZ, and GB: critical revision. All authors contributed to the article and approved the submitted version.

## Conflict of Interest

The authors declare that the research was conducted in the absence of any commercial or financial relationships that could be construed as a potential conflict of interest.
